# ORAL FRAILTY INDEX-8 QUESTIONNAIRE’S VALIDITY & ASSOCIATION WITH SARCOPENIA & PHYSICAL PERFORMANCE IN OLDER ADULTS

**DOI:** 10.1093/geroni/igae098.4037

**Published:** 2024-12-31

**Authors:** Herb Howard Hernandez, Celestine Lim, Jun Pei Lim, Jia Qian Chia, Audrey Yeo, Cai Ning Tan, Wee Shiong Lim, Justin Chew

**Affiliations:** Tan Tock Seng Hospital, Singapore, Singapore; Tan Tock Seng Hospital, Singapore, Singapore; Tan Tock Seng Hospital, Singapore, Singapore; Tan Tock Seng Hospital, Singapore, Singapore; Tan Tock Seng Hospital, Singapore, Singapore; Tan Tock Seng Hospital, Singapore, Singapore; Tan Tock Seng Hospital, Singapore, Singapore; Tan Tock Seng Hospital, Singapore, Singapore

## Abstract

Oral frailty (OF) is the age-related decline in oral function, and is associated with negative outcomes, including aspiration, malnutrition, and physical frailty. Despite its importance, validated tools for OF assessment are limited, preventing early detection and intervention. The Oral Frailty Index-8 (OFI-8) is a questionnaire assessing tooth loss, chewing, swallowing and oral health behaviours. We aim to evaluate OFI-8’s construct validity and determine its associations with muscle strength, physical performance, sarcopenia, and functional outcomes. We conducted a cross-sectional analysis of 300 community-dwelling older adults (mean age 67.4+/-7.1years), using exploratory factor analysis (EFA) to determine OFI-8’s factor structure. We assessed OFI-8’s correlations with dual-energy X-ray absorptiometry (DXA)-derived appendicular lean mass corrected for height (ALM/ht2), maximum handgrip strength and usual gait speed. We then examined the associations of OFI-8 with outcomes of sarcopenia (self-reported questionnaire,SARC-F), physical performance (Short Physical Performance Battery,SPPB) and IADL function (Lawton scale) using multiple linear regression adjusted for age and sex. EFA revealed a 3-factor structure for OFI-8 (Factor-1:Oral and Swallowing Problems; Factor-2:Dental Care; Factor-3:Dietary and Social Habits). Higher OFI-8 scores, indicating OF, demonstrated weak but significant correlations with low lean mass(r=-0.119,P=0.038), impaired handgrip strength(r=-0.218,P=0.001), and slow gait speed(r=-0.203,P=0.004), and was associated with sarcopenia (Beta=0.044,95%CI 0.0012 to 0.089,P=0.044), impaired physical performance (Beta=-0.088,95%CI -0.156 to -0.021,P=0.011), and IADL impairment (Beta=-0.080,95%CI -0.15 to -0.010,P=0.026). Our results provide preliminary evidence for the factor structure and validity for the OFI-8, as well as its association with sarcopenia and functional impairment. Further research is required to evaluate its predictive validity for longitudinal outcomes.

